# Successful conservative surgical management of first-trimester placenta accreta: Case report

**DOI:** 10.1016/j.ijscr.2024.110172

**Published:** 2024-08-13

**Authors:** Nesrine Souayeh, Hana Smida, Hadhami Rouis, Amira Lika, Chaouki Mbarki, Hajer Bettaieb

**Affiliations:** Department of Gynecology and Obstetrics, Ben Arous Regional Hospital, Ben Arous, Tunisia; Faculty of Medicine, University of Tunis el Manar, Tunis, Tunisia

**Keywords:** Placenta accreta, First trimester, Diagnosis, Conservative management

## Abstract

**Introduction and importance:**

Placenta accreta spectrum in the first trimester is a rare but life-threatening condition. Its diagnosis and management remain challenging due to the lack of diagnostic criteria and therapeutic guidelines. This case report emphasizes the importance of early diagnosis of first trimester placenta accreta to perform fertility-sparing management.

**Case presentation:**

A 29-year-old gravida 2 para 1 woman, with history of cesarean delivery, presented with abnormal uterine bleeding. On physical examination, she had minimal vaginal bleeding with normal haemodynamic parameters. An endovaginal ultrasound revealed a non-viable fetus and a low implanted gestational sac. Cesarean scar pregnancy (CSP) was suspected. The patient underwent an ultrasound-guided uterine dilatation and curettage, complicated with massive bleeding. Before an emergency laparotomy was carried out, bleeding was controlled with a Foley catheter balloon. Conservative management was performed with bilateral hypogastric artery ligation followed by the placenta accreta niche resection. Pathology confirmed first-trimester placenta accreta.

**Clinical discussion:**

Placenta accreta spectrum disorders can occur even in the first trimester. Traditionally, hysterectomy has been the treatment of choice, but conservative management is possible with careful case selection and monitoring. Careful preoperative planning, including multidisciplinary consultation, is key to improving maternal outcomes. Maintaining high index of suspicion for placenta accreta spectrum disorders, and early diagnosis through ultrasonography, is crucial in the first trimester to perform fertility-sparing surgical management.

**Conclusion:**

Placenta accreta spectrum incidence is increasingly rising. First-trimester placenta accreta should be suspected in high-risk situations. Conservative management can be offered in selected cases.

## Highlights

• Placenta accreta spectrum (PAS) incidence is rising, and first-trimester diagnosis is challenging due to low accuracy.
• This paper reports a successful conservative surgical management case of first-trimester PAS.
• Early diagnosis is crucial, and conservative management can preserve future pregnancy potential.
• Foley catheter balloon to control the bleeding, can buy time to perform an emergency laparotomy

## 1 Introduction

Placenta accreta spectrum (PAS) is a life-threatening condition, that can be responsible for severe obstetric hemorrhage, resulting in multiple transfusions, hysterectomy, and damage to adjacent organs [1,2]. The incidence of PAS has been increasing over the years, from 1 in 4000 births in 1970 to 1 in 533 births in 2005, due to an increasing number of cesarean deliveries [1,3]. This disorder is typically diagnosed in the second and third trimester of pregnancy. The diagnosis of PAS in the first trimester is rare and challenging giving the low accuracy of diagnostic tools at this term [4].

A prenatal diagnosis is essential for organizing the best management for PAS. Since ultrasonography (US) is readily available, non-invasive, and highly sensitive, it is the preferred method for evaluating and diagnosing suspected PA. When US is unconclusive, magnetic resonance (MR) is used as a supplement [5].

The treatment of PAS in the first trimester is not well codified due to its rarity. Hysterectomy is often the primary treatment or a last resort if conservative care fails [6]. We report a case of a successful conservative surgical management of first-trimester placenta accreta.

This work has been reported in line with the SCARE criteria [7].

## 2 Case presentation

**Fig. 1:**
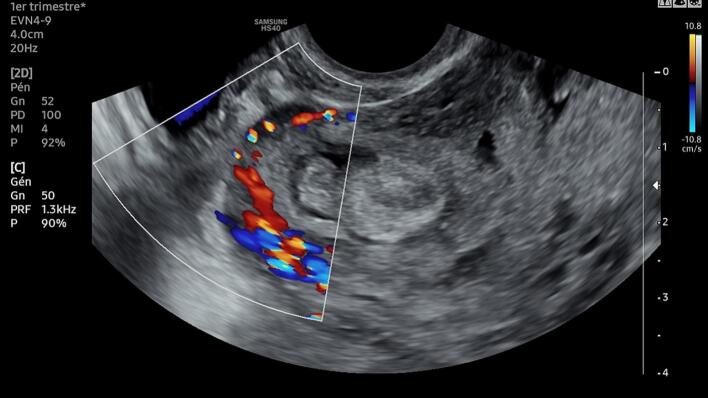
Endovaginal ultrasound showing a low implanted pregnancy with a thin uterine-placental interface, and a turbulent blood flow.

**Fig. 2:**
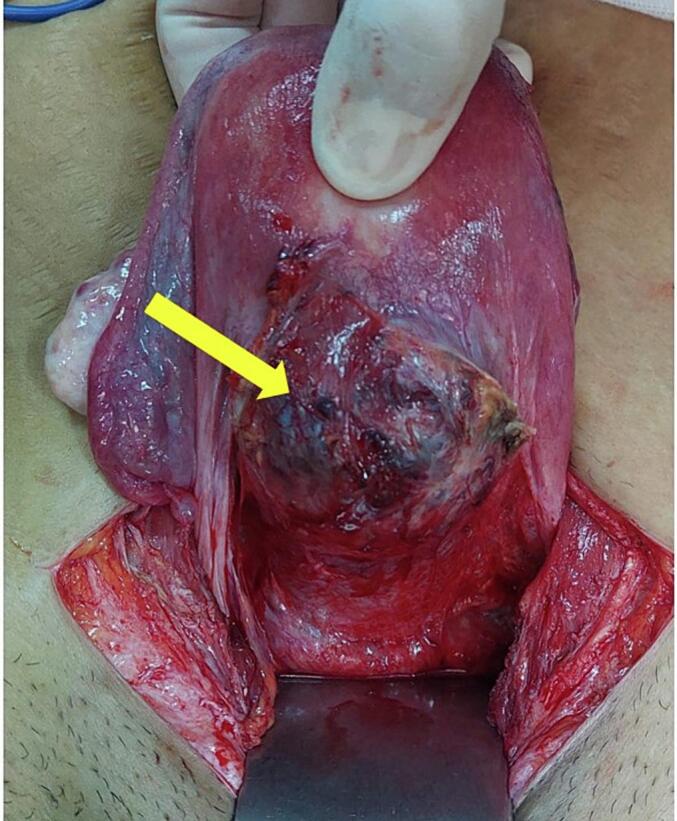
Per-operative findings showing abnormally exaggerated isthmic vascularization (yellow arrow).

**Fig. 3:**
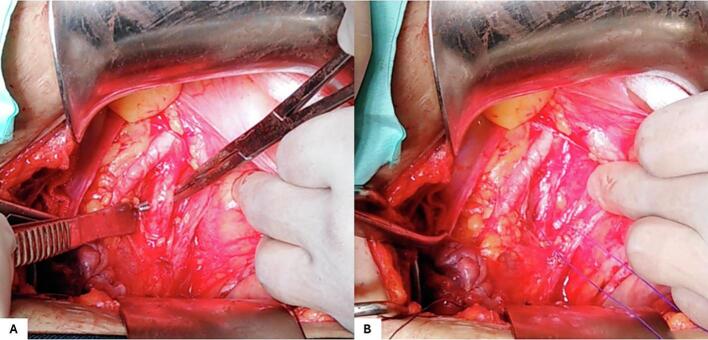
Hypogastric artery ligation procedure. A: Dissection and mobilization of the internal iliac artery. B: Ligation of the internal iliac artery two centimeters below the bifurcation.

**Fig. 4:**
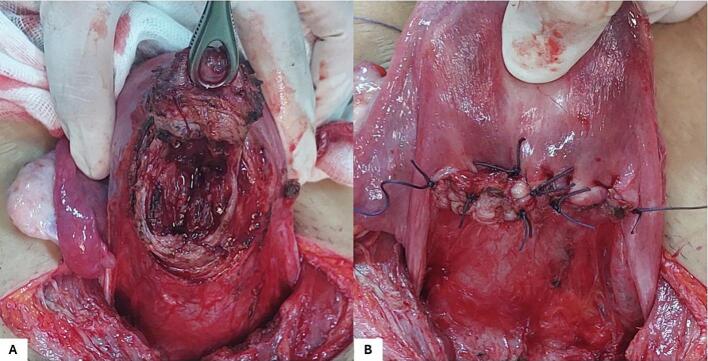
Surgical ablation of the placenta accreta niche A: wedge-shaped resection of the placenta accreta niche. B: Final result after the resection-suture of the placenta accreta niche.

A 29-year-old gravida 2 para 1 presented to our department with abnormal uterine bleeding with a 10-week amenorrhea period. The patient's first pregnancy was uneventful, and the delivery was scheduled at 39 gestation weeks (GW) by cesarean section for fetal macrosomia. The current pregnancy was not planned, and the patient didn't undergo a prior ultra-sonography examination. On admission, the patient presented on physical examination minimal vaginal bleeding, a 120/70 mmHg blood pressure and a pulse rate at 82 bpm. Endovaginal ultrasound found a low implanted gestational sac with a thin uterovesical interface, and loss of the clear zone. The color flow Doppler showed intra-placental turbulent blood flow (Fig. 1). The fetus had no heartbeat. The diagnosis of cesarean scar pregnancy (CSP) was suspected. We then decided to perform an ultrasound-guided uterine dilatation and curettage. During the surgery, the patient presented massive bleeding with an accelerated pulse rate up to 110 bpm and dropped blood pressure down to 80/60 mmHg. We inserted a Foley catheter balloon to control the bleeding, time to perform an emergency laparotomy. The pelvic cavity inspection found no hemoperitoneum. The lower uterine segment was bulging, and some area revealed placental tissue through the serosa with abnormally dilated vessels (Fig. 2). Given the patient's age and low parity, we decided to go for conservative management. Because of the low implantation of the pregnancy, we performed a bilateral hypogastric artery ligation in order to stop the bleeding. After retracting the intestines and other abdominal contents, we opened the retroperitoneal space between the round and the infundibular ligament and identified the bifurcation of the common iliac artery into the internal and external iliac arteries. We then carefully mobilized the internal iliac artery by dissecting the surrounding tissues, being cautious to avoid injury to nearby structures such as the ureter and veins (Fig. 3A). We used an absorbable suture to place ligatures around the internal iliac artery two centimeters below the bifurcation (Fig. 3B). Afterwards, we grasped the placenta accreta niche with atraumatic forceps and performed a wedge-shaped resection using the electric scalpel, passing through healthy tissue (Fig. 4A). The uterine margins were then sutured with separate absorbable sutures to re-establish continuity (Fig. 4B). Per operative hemoglobin dropped from 11.2 g/dl to 8.8 g/dl and the patient did not require blood transfusion. On pathology examination, we noticed the presence of extra villous trophoblastic cells in the superficial myometrium and occasional chorionic villi attached to the myometrium, confirming the diagnosis of first-trimester placenta accreta. The post-operative course was favorable, and the patient was discharged on oral contraception.

## 3 Discussion

Although the incidence of placenta accreta has kept rising these last decades, it is still rare in the first trimester. The largest systematic review including 52 cases of PAS in the first trimester was published in 2022 [1], with an overlap of 17 cases with the previous review of 23 cases published in 2019 [6], highlighting its rarity. The main two risk factors of placenta accreta are previous cesarean delivery and placenta previa. In a large prospective cohort including 30,132 women with history of cesarean delivery, Silver et al. showed that the risk of developing PAS was positively correlated to the number of cesarean scars (rising from 0.24 % at first cesarean, up to 6.74 % in the sixth cesarean) [8]. In women with placenta previa, this risk was at 3.3 %, 11 %, 40 %, 61 %, and 67 % for the first, second, third, fourth, and fifth previous cesareans, respectively [8]. Our patient had both risk factors i.e. a history of cesarean delivery and a low implanted placenta.

Despite its low incidence, PAS should be suspected in case of abnormal uterine bleeding following first-trimester surgical evacuation in high-risk cases. In their review of 23 cases, Wang et al. reported vaginal bleeding in 22 patients (95.65 %), ranging from intermittent or irregular bleeding to massive bleeding after surgical abortion [6].

The diagnosis and treatment of PAS in early pregnancy is challenging due to the few cases described in the literature, limited by experience, and poorly defined diagnostic criteria. Early diagnosis of PAS can help to optimally plan treatment and improve maternal outcome [9]. Many authors tried to identify ultrasonographic diagnostic features of PAS in the first trimester. According to D'Antonio et al., the most frequent ultrasound feature in the first trimester of pregnancy was low implanted gestational sac (82.4 %) followed by placental lacunae (46.0 %) [10]. Gali et al. found that the following three sonographic features 1) crossover sign (COS), 2) implantation of the gestational sac on the scar vs in the niche of the C-section, 3) position of the center of the gestational sac below vs above the midline of the uterus were associated with the severest form of PAS disorder in respectively 79.6 %, 94.4 % and 100 % of cases [11]. According to Rahimi-Sharbaf et al., ultrasonography had a sensitivity and specificity for detecting PAS in the first trimester of 41 % and 88 % respectively [4].

First-trimester PAS management is not well codified. The standard approach remains to be hysterectomy [6]. However, conservative management can be considered in selected patients of childbearing age with a desire for uterine preservation. Uterine artery embolization (UAE) and cytotoxic therapy with methotrexate (MTX) have been introduced recently as conservative measures for the treatment of post-abortal and postpartum abnormal placentation [6,12,13]. Wang et al. reported a case of placenta augmentation after first-trimester abortion, which was successfully treated by laparoscopic hysterotomy and removal of placental tissue [6]. In case of bleeding occurrence, a laparoscopic hysterectomy could be performed. Laparotomy should be performed as a last resort. In our case, given the absence of UAE, we performed a bilateral hypogastric artery ligation to reduce blood flow and facilitate the resection of placental tissue.

## 4 Patient's perspective

The patient was grateful we could save her uterus.

## 5 Conclusion

Placenta accreta spectrum disorders, though rare in the first trimester, should be considered in high-risk patients. First-trimester ultrasound could detect abnormal placentation in many cases despite its low sensitivity and specificity. Early diagnosis and conservative management should be tailored on a case-by-case basis, considering the patient's desire for future pregnancy, the severity of the bleeding, and the available local expertise.

## Ethical statement

This case report was approved by the local ethics committee of our institution under the number 02/2024. Written informed consent was obtained from the patient for publication and any accompanying images. A copy of the written consent is available for review by the Editor-in-Chief of this journal on request.

## Funding

This research did not receive any specific grant from funding agencies in the public, commercial, or not-for-profit sectors.

## Author contribution

Drafting the article: Nesrine Souayeh, Hajer Bettaieb.

Acquisition of data: Hana Smida, Hadhami Rouis.

Revising the article: Nesrine Souayeh, Hajer Bettaieb, Chaouki Mbarki.

All the authors have read and agreed to the final manuscript.

## Guarantor

Nesrine Souayeh.

## Registration of research studies

Not applicable.

## Consent

Written informed consent was obtained from the patient for publication and any accompanying images. A copy of the written consent is available for review by the Editor-in-Chief of this journal on request.

## Declaration of competing interest

Authors declared they have no conflicts of interest.
